# The Analysis of Risk Factors for Hemorrhage Associated with Minimally Invasive Percutaneous Nephrolithotomy

**DOI:** 10.1155/2019/8619460

**Published:** 2019-01-30

**Authors:** Xiangjun Meng, Juan Bao, Qiwu Mi, Shaowei Fang

**Affiliations:** ^1^Department of Urology, Dongguan people's Hospital, Dongguan 523059, Guangdong, China; ^2^Dongguan people's Hospital, Dongguan 523059, Guangdong, China

## Abstract

**Objective:**

This study investigated the risk factors for bleeding during minimally invasive percutaneous nephrolithotomy, so as to prevent the occurrence of bleeding and improve the surgical effect.

**Patients and Methods:**

The data of 396 patients who underwent percutaneous nephrolithotomy by an experienced surgeon between May 2014 and December 2017 were retrospectively analyzed. To identify the risk factors for bleeding during percutaneous nephrolithotomy, each group was stratified according to the decrease in median hemoglobin. Age, gender, body mass index, stone size, operation time, stone type, degree of hydronephrosis, number of accesses, puncture guidance, underlying disease (diabetes; hypertension), and previous surgical history were evaluated. Univariate analysis was performed to calculate the potential factors. In order to determine the independence of each factor, we finally selected stone size, staghorn stone, degree of hydronephrosis, and operation time. Multivariate logistic regression analysis was used to identify the risk factors for bleeding during minimally invasive percutaneous nephrolithotomy.

**Results:**

A total of 396 patients were successfully treated with percutaneous nephrolithotomy. The univariate analysis demonstrated that the potential risk factors for bleeding during percutaneous nephrolithotomy included stone size, type of stone, operative time, and degree of hydronephrosis. According to the previous studies, stone size, staghorn stone, degree of hydronephrosis, and operation time were ultimately selected. Multivariate logistic regression analysis was used to identify the risk factors for bleeding during percutaneous nephrolithotomy. According to the outcome of logistic regression analysis, stone size, staghorn stone, operation time, and degree of hydronephrosis were the risk factors for bleeding during minimally invasive percutaneous nephrolithotomy.

**Conclusions:**

Percutaneous nephrolithotomy is an effective method for the treatment of upper urinary calculi with few complications. According to the results achieved by an experienced surgeon, the size of stone, staghorn stone, operation time, and degree of hydronephrosis were associated with the bleeding during minimally invasive percutaneous nephrolithotomy.

## 1. Introduction

Percutaneous nephrolithotomy for the treatment of renal and ureteral calculi is effective, minimally invasive, and repeatable. Michel et al. reported that the success rate of percutaneous nephrolithotomy was more than 90%, but bleeding was still a common and serious complication, and the decrease in the hemoglobin level was 2.1-3.3 g/dl [1, 2]. Although most bleeding can be treated conservatively, severe bleeding that requires renal arteriography and selective embolization still occurs (approximately 0.8%) [3]. Therefore, it is important to identify the risk factors that may affect the incidence of bleeding and take active measures to reduce the occurrence of hemorrhage rather than take remedial measures after the occurrence of bleeding. Traditionally, diabetes, staghorn calculi, and the number of access tracts may be risk factors for bleeding during minimally invasive percutaneous nephrolithotomy [4, 5]. To identify factors influencing bleeding during minimally invasive percutaneous nephrolithotomy, the date of 396 patients were retrospectively analyzed in our study. All procedures were performed by a single surgeon.

## 2. Patients and Methods

This study protocol was approved by the institutional review committee, and patients provided written informed consent to publish details information in this manuscript.

From May 2014 to December 2017, 396 patients (191 male; 205 female) with urinary calculi treated with minimally invasive percutaneous nephrolithotomy were retrospectively enrolled in this study. The mean age was 42.5±13.1 years (range 12~73). There were 186 right kidney stones and 210 left kidney stones. The types of stones included staghorn stones (83 cases, 21%), renal pelvis stones (145 cases, 36.6%), calyceal stones (110 cases, 27.8%), and upper ureteral stones (58 cases, 14.6%) (Table 1). Surgeries were performed by a single surgeon with 10 years of experience. Patients with long-term antiplatelet or anticoagulation treatment were excluded from our study.

All patients underwent preoperative intravenous urography, urinary ultrasonography, and computed tomography. Patients underwent the placement of an ipsilateral ureteral catheter under general anesthesia and turned into a full prone position. The access site was selected with the aid of CT and intravenous urography. Puncture was randomly performed under fluoroscopic or ultrasound guidance. The channel was dilated with serial dilators up to 18-Fr, and an 18-Fr Amplatz sheath (COOK Medical Inc., Bloomington, USA) was inserted into the renal calyx over the inflated Nephromax. A Wolf nephroscope (11.5F) was used in percutaneous nephrolithotomy, and stones were fragmented with a pneumatic lithotripsy device (Hawk Medical Inc., Shenzhen, China). Stone fragments were removed by forceps via Amplatz sheath. According to the distribution of stones, the surgeon created additional channels to clear them.

Urinary ultrasound was used to evaluate the degree of hydronephrosis, which was graded as nil, mild, moderate, or severe. The degree of hydronephrosis was graded by ultrasonic grading method: (1) nil hydronephrosis: the renal morphology, renal parenchyma, and renal collection system remained unchanged; (2) mild hydronephrosis: the morphology and parenchyma of kidney remained unchanged, only pyelomegaly appeared; (3) moderate hydronephrosis: the morphology and parenchyma of kidney remained unchanged, and both pelvis and calyx were dilated; (4) severe hydronephrosis: the renal volume increased, the renal parenchyma was compressed and thinned, and the hydronephrosis was palette-like. The size of the stone was measured by computed tomography.

Urine routine and urine culture were used to evaluate whether or not the urinary tract infection merged. Patients with urinary tract infection were treated with antibiotics before the procedures.

The mean decrease in hemoglobin was 2.19 ±0.56 g/dl, and the median decrease in hemoglobin was 2.23 g/dl. The patients were stratified according to the median decrease in hemoglobin, in Group 1 (198 patients), the mean decrease in hemoglobin was less than the median decrease in hemoglobin, and in Group 2 (198 patients), the mean decrease in hemoglobin was greater than the median decrease in hemoglobin. Factors influencing bleeding during minimally invasive percutaneous nephrolithotomy were identified by comparison of age, sex, body mass index, stone size, stone type, puncture guidance, duration of operation, degree of hydronephrosis, underlying disease, previous surgical history, and number of accesses.

SPSS 17.0 (SPSS, Chicago, IL, USA) was used for statistical analysis. Data were reported as mean plus or minus the standard deviation (SD) or the median and range. Continuous variables were compared by the paired nominal variables, and Pearson's test were used for analysis. Wilcoxon test and Chi-square test were used for univariate analyses. Then, multivariate binary logistic regression was used for multivariate analysis. A p-value of < 0.05 was considered statistically significant.

## 3. Results

All patients were successfully treated with minimally invasive percutaneous nephrolithotomy. The comparisons between lesser and greater bleeding groups for clinical and perioperative factors were summarized in Table 2. The median decrease in hemoglobin (2.23 g/dl) was considered as a cutoff point. According to the median decrease in hemoglobin, the patients were divided into Group 1 (1.78 ±0.39 g/dl) and Group 2 (2.60±0.37 g/dl), and there was a statistically significant difference between the two groups (p < 0.001).

The mean age of Group 1 was 42.3±13.4 years and that of Group 2 was 42.7±12.8 years, with no significant difference between the two groups (p = 0.71); no statistically significant difference was found between the two groups in gender (p = 0.56). Group 1 included 98 left kidney stones and 100 right kidney stones, and Group 2 included 112 left kidney stones and 86 right kidney stones; no significant difference was found between the two groups (p = 0.159).

The mean stone size was 504.7±185.6 mm^2^ in Group 1 and 734±285.6 mm^2^ in Group 2, respectively, indicating a significantly larger stone size in Group 2 (p < 0.001). Group 2 included 61 staghorn stones, and Group 1 included 22 staghorn stones. Thus, there were more staghorn stones in Group 2, and a significant difference was found between the two groups (p < 0.001). In Group 1, a total of 97 cases were guided by ultrasound guidance and 101 cases by fluoroscopic guidance in Group 1. In Group 2, a total of 89 cases were guided by ultrasound guidance and 109 cases by fluoroscopic guidance; no significant difference was found between the two groups (p = 0.421).

The mean operation time was 66.7 ±17.5 min in Group 2 and 61.2±20.9 min in Group 1, and a significant difference was found between the two groups (p = 0.005). In terms of preoperative hydronephrosis, a significant difference was found between the two groups (p < 0.001). According to the distribution of calculi, a total of 51 patients were treated with multiple-channels procedures in Group 1 and 68 patients in Group 2; no significant difference was found in number of access between the two groups (p = 0.062).

Thirteen cases were treated by open surgery in Group 1 and 10 cases in Group 2; no significant difference was found between the two groups (p = 0.519). There were no significant differences between the two groups in terms of the previous history of percutaneous nephrolithotomy (p = 0.277). Although a history of ipsilateral ESWL was present in 26 (13.1%) and 30 (15.2%) patients, respectively, there was no significant difference between the two groups (p = 0.564). There were 33 patients with diabetes mellitus in Group 1 and 23 patients in Group 2; no significant difference was found between the two groups (p = 0.149). Additionally, no significant difference was found between the two groups in terms of hypertension (p = 0.336).

The univariate analysis demonstrated the potential risk factors for bleeding during minimally invasive percutaneous nephrolithotomy, including stone size (p < 0.001), stone type (p < 0.001), operative time (p = 0.005), and degree of hydronephrosis (p < 0.001). According to previous studies reported in the literature, as well as clinical experience, we finally selected stone size, staghorn stone, operation time, and hydronephrosis to identify the independence of various factors. Multivariate logistic regression analysis was used to identify the risk factors for bleeding during minimally invasive percutaneous nephrolithotomy. The outcome of multivariate logistic regression analysis showed that there were four risk factors associated with bleeding during minimally invasive percutaneous nephrolithotomy, including stone size (p < 0.001), staghorn stone (p = 0.008), operation time (p = 0.013), and degree of hydronephrosis (p < 0.001) (Table 3).

## 4. Discussion

With the improvement of surgical techniques, minimally invasive surgical methods have rapidly developed in recent years. Percutaneous nephrolithotomy is a minimally invasive and reproducible method for the treatment of kidney stones, and the procedure is repeatable. Although percutaneous nephrolithotomy has become the standard treatment for kidney stones, there is still a potential risk of complication [6–8].

The incidence of complications associated with percutaneous nephrolithotomy is 15% [9]. Bleeding is one of the most serious complications. Michel et al. reported that the success rate of percutaneous nephrolithotomy was more than 90%, but bleeding was a common and serious complication, and the decrease in hemoglobin level was 2.1-3.3 g/dl [1, 2, 10]. In a Global Study analysis, the incidence of bleeding was 9.4% [11]. Although most bleeding can be treated conservatively, there is still severe bleeding (approximately 0.8%) that requires renal arteriography and selective embolization [3]. Therefore, it is important to identify the risk factors that may affect the incidence of bleeding and take active measures to reduce the occurrence of hemorrhage rather than take remedial measures.

Because diabetes can lead to atherosclerosis and microangiopathies, patients with diabetes mellitus are prone to bleeding compared with patients without diabetes mellitus [4]. Kukreja and colleagues reported that the relationship between hypertension and bleeding during percutaneous nephrolithotomy was explained with arteriosclerosis [12]. In this study, there were 33 patients with diabetes (Group 1) compared with 23 cases (Group 2), and no significant difference was found between the two groups (t = 2.08, p = 2.08). Although hypertension was correlated with atherosclerosis, there was no significant difference in hypertension in our study (t=0.925; p = 0.336). Therefore, we believe that diabetes and hypertension are not risk factors for bleeding during percutaneous nephrolithotomy.

Previous studies have shown that females were relatively prone to bleeding due to their susceptibility to urethral infection, but this view has not been shared by other researchers [13–15]. To identify whether or not gender was a risk factor for bleeding, gender was observed in our study, but the data did not show any relationship between gender and bleeding, so we did not conclude that gender was a risk factor for bleeding during percutaneous nephrolithotomy.

In the literature, stone size was a risk factor for bleeding during percutaneous nephrolithotomy [12, 16]. Since the goal of percutaneous nephrolithotomy is to remove stones, relieve obstruction, and protect renal function, it is necessary to reach the entire calyx, which may tear the neck of the calyx and result in bleeding [17, 18]. To remove stones, the operation time is relatively prolonged due to large calculi, and the blood loss would increase compared with that of small calculi, which has been confirmed in our study. Through univariate analysis or multivariate logistic regression analysis, we found that the size of stone and the duration of operation were risk factors for bleeding during percutaneous nephrolithotomy.

The type of stone is a risk factor for bleeding during percutaneous nephrolithotomy, which has been confirmed by many researchers [19]. According to the distribution of stones in the renal pelvis and calyx, the stones distributed in the calyx and more than two calyxes are defined as staghorn calculi. Turna et al. considered that the type of stone could greatly influence bleeding during percutaneous nephrolithotomy and emphasized that an incomplete or complete antler stone was a high risk factor for bleeding [15, 20]. Because the cortex of kidneys with staghorn calculi is relatively thicker, the channel of percutaneous nephrolithotomy is longer; patients with staghorn calculi are prone to bleeding. According to the outcomes of univariate analysis, the type of stone may affect hemorrhage associated with percutaneous nephrolithotomy. In the multivariate analysis, staghorn calculi were a risk factor for hemorrhage associated with percutaneous nephrolithotomy.

Yesil reported that kidneys adhered to surrounding tissues after open surgery, the position of kidneys was relatively fixed, the range of activity decreased, and the incidence of calyx laceration increased [21, 22]. The history of open surgery, percutaneous nephrolithotomy, and extracorporeal shock wave lithotripsy was reviewed in our study; according to univariate analysis, we did not find that the possibility of bleeding during percutaneous nephrolithotomy increased.

It is generally believed that patients with stones with nil and mild hydronephrosis are prone to severe bleeding due to the relatively thick renal cortex [23]. As the degree of hydronephrosis increases, the distribution of renal vessels will be relatively sparse; therefore, the possibility of renal vessel injury decreases during percutaneous nephrolithotomy. According to the results of univariate analysis, we found that there was greater bleeding in kidneys with nil and mild hydronephrosis. Then, the degree of hydronephrosis was analyzed by multiple variate logistic regression analysis, and the results showed that the degree of hydronephrosis was risk factor for bleeding during percutaneous nephrolithotomy.

In this study, there were several limitations. First, in our study, 18F channel was routinely used, and the dilation of channel was performed using Amplatz dilators in all cases. Therefore, this factor was not analyzed in this study. Second, due to the retrospective nature of our study, selection bias exists.

## 5. Conclusions

According to the results achieved by a single experienced surgeon, stone size, staghorn stone, operation time, and degree of hydronephrosis are associated with hemorrhage during percutaneous nephrolithotomy. It is important to identify the risk factors that may affect the incidence of bleeding and take active measures to prevent the occurrence of hemorrhage before performing percutaneous nephrolithotomy rather than taking remedial measures.

## Figures and Tables

**Table 1:** Patient demographics, stone characteristics, and surgical outcomes.

Variables	Mean (SD) or n / N	Median (range)

No. of patients (n)	396	
Age (years)	42.5(13.1)	42.5 (12~73)
Male:female (n)	191 /205	
BMI(kg/m^2^)	24.7(2.6)	25.3 (18.3~29.6)
Right/left side (n)	186 /210	–
Stone size (mm^2^)	617.3(266.5)	560 (230~1780)
Stone type		
Staghorn	83 (21)	
Renal pelvis	145 (36.6)	
Calyceal	110 (27.8)	
Upper ureter	58 (14.6)	
Drop in hemoglobin		
(g/dl)	2.19 (0.56)	2.23(0.94–5.69))

BMI = body mass index.

**Table 2:** Comparison of clinical and perioperative factors between less and greater bleeding groups.

Variable	Group 1	Group 2	t/*χ*^2^	P* value*

Decrease in hemoglobin	1.78 ±0.39	2.60 ±0.37	-21.5	0.000
(g/dl)				
Age, years	42.3 ±13.4	42.7 ±12.8	-0.372	0.710
Gender			3.651	0.56
Male	105(53)	86(43.4)		
Female	93(47)	112(56.6)		
BMI (kg/m^2^)	24.7 ±2.6	24.6 ±2.5	0.344	0.731
Sides			1.987	0.159
Right	100(50.5)	86(43.4)		
Left	98(49.5)	112(566)		
Stone size (mm^2^)	504.7 ±185.6	734 ±285.6	9.476	0.000
Stone type			38.9	0.000
Staghorn	22(11.1)	61(30.8)		
Renal pelvis	71(35.9)	74(37.4)		
Calyceal	59(29.8)	51(25.8)		
Upper ureter	46(23.2)	12(6.1)		
Puncture guidance			0.649	0.421
ultrasound-guidance	97(49)	89(44.9)		
fluoroscopic-guidance	101(51)	109(55.1)		
Duration of operation (min)				
mean ±SD	61.2 ±20.9	66.7 ±17.5	-2.812	0.005
Degree of hydronephrosis			65.45	0.000
Nil	20(10.1)	59(29.8)		
Mild	79(39.9)	110(55.6)		
Moderate	82(41.4)	19(9.6)		
Severe	17(8.6)	10(5.1)		
Previous surgery				
Previous open surgery	13 (6.6)	10 (5.1)	0.415	0.519
Previous PCNL	18(9.6)	13 (6.6)	1.182	0.277
History ESWL	26(13.1)	30 (15.2)	0.333	0.564
Underlying disease				
Diabetes mellitus	33(16.7)	23(11.6)	2.08	0.149
Hypertension	28(14.1)	35(17.7)	0.925	0.336
Number of accesses			3.472	0.062
Single	147(74.2)	130(65.7)		
Multiple	51(25.8)	68(34.3)		

BMI = body mass index; PCNL = percutaneous nephrolithotomy; ESWL = extracorporeal shock wave lithotripsy.

**Table 3:** Outcomes of multivariate binary logistic regression analysis: factors affecting total blood loss.

Factors	B	Wals	P-value	OR	95%CI

Stone size (mm^2^)	0.975	28.766	<0.001	2.652	1.857-3.788
Staghorn	0.378	7.092	0.008	1.459	1.105-1.926
NO					
YES					
Duration of operation	0.676	6.177	0.013	1.965	1.154-3.348
Degree of hydronephrosis					
	-1.366	45.749	<0.001	0.255	0.172-0.379

According to median drop in hemoglobin; OR=odds ratio; CI=confidence interval.

## Data Availability

The data used to support the findings of this study are available from the corresponding author upon request.

## Abbreviations

PCNL: Percutaneous nephrolithotomy
ESWL: Extracorporeal shock wave lithotripsy
BMI: Body mass index
CT: Computer tomography
OR: Odds ratio
CI: Confidence interval.
